# Editorial: Meditative movement for mental and physical health

**DOI:** 10.3389/fpsyg.2023.1238633

**Published:** 2023-07-04

**Authors:** Russell Sarwar Kabir, Hyun-Jeong Yang

**Affiliations:** ^1^Graduate School of Humanities and Social Sciences, Hiroshima University, Higashihiroshima, Japan; ^2^Korea Institute of Brain Science, Seoul, Republic of Korea; ^3^Department of Integrative Healthcare, University of Brain Education, Cheonan, Republic of Korea

**Keywords:** meditative movement, yoga, qigong, Dohsa-hou, executive functions, gut-brain axis, physical activity, aerobic exercise

Agents of therapeutic change in mental and physical health promotion contexts, such as meditation and physical exercise, might enhance the regulation of internal states in ways that provide beneficial effects to human health and functioning. Behavioral health practices such as exercise, yoga, tai chi, qigong, Dohsa-hou, and other movement-related approaches offer platforms replete with the protective factors requisite for regulating physical activity and balancing mental states.

Coupling meditative elements into body movement processes is likely to have the advantages of inducing concentration more easily even among novice practitioners as it encourages focus on specific parts or senses of the body. Despite potential contributions to individual, social, and environmental categories of mental health and wellbeing, practices that invoke movement for these purposes are packaged in ways that require close inspection and evaluation of the specificity, duration, conditions, and limitations of their effects. For example, *static* meditation may be somewhat less accessible to beginners or those who have difficulty maintaining their concentration, and physical exercise at higher thresholds of intensity may not be accessible to groups with certain characteristics. These points suggest that dynamic meditative and movement-integrated applications might need to be introduced to users in cohesive or streamlined ways, in addition to leveraging their unique features and drawing upon the evidence base of their therapeutic or health-promoting elements.

The field of *meditative movement* research is relatively less developed in comparison to conventional meditation research, and far less developed than experimental and effectiveness studies of exercise and physical activity. Furthermore, while the movement and attentional processes that are common to contemplative practices play a role in the control and maintenance of physical and mental health, advances in the field tend to emphasize either physical exercise or psychosocial interventions and their effects on applied outcomes in isolation. This might be due to the dynamism inherent to cognition and movement and their related factors, or possibly, a lack of interdisciplinary exposure and coordination. In this effort, we aimed to improve the understanding of the effects of meditative movement on mental and physical health with research that highlights their potential to connect to underlying physiological, cognitive, or regulatory processes.

## Psychophysiological proposals

Addressing the need for deeper descriptions about dynamics, Ningthoujam et al. provided research synthesis on routes for movement, relaxation, and awareness to intersect through cyclic meditation and influence the regulation of the gut-brain axis. Linking processes like afferent and efferent vagal nerve stimulation to cognitive functions, as well as connecting stress response effects to changes in gut microbiota, the proposal puts forth an original research program and agenda for future systematic research in psychophysiology.

## Meditative movement for mental health and executive functions

In two studies recruiting healthy participants from the United States and Japan, and one study of clinical outcomes in the United States, three meditative movement practices were evaluated for their effects on mental health. Fritz and O'Connor investigated the effects of a 6-week pilot yoga program on psychological outcomes and executive functioning for women screening positive for ADHD, finding feasible implementation but null findings for effects from the yoga intervention on the study variables for the length and intensity of yoga that was implemented. Fujikawa et al. compared indices from the dual mechanisms of cognitive control, measured via AX-CPT and a modified Stroop task in an experiment with conditions manipulating attention to the shoulder movement, whose results supported some changes reflecting the promotion of reactive control and the balance of control modes in the Dohsa-hou task condition that included verbal feedback from the practitioner. In a 12-week qigong program for breast cancer survivors with persistent post-surgical pain, Quixadá et al. showed relationships between posture and mood in terms of vertical head values, as well as associations between vertical spine angles and changes that indicated improvements in fatigue, anxiety, and pain severity.

## Physical exercise for mental health and executive functions

In studies from Switzerland and China, physical exercise was examined for its effects on mindfulness, resilience, and other outcomes. Tendencies among respondents in the survey-based research contribution from Philippe et al. offered support for mindfulness practice as a coping tool based on initial levels of resilience during the COVID-19 pandemic, and indicated that physical activity might be associated with decreasing depression and maintaining resilience over time. Similarly, with undergraduate students, Zhou and Bai showed some improved indicators of inhibitory control from performance on an antisaccade task after 15 min of moderate intensity exercise regardless of cutoff thresholds for mobile phone addiction.

## Future directions

These findings show specific effects for practices on psychological outcomes and extend support for the effects of aerobic exercise on cognitive processes. Our Research Topic clarified new insights from theorized points of cyclic meditation and empirical studies of approaches that integrate physical movement, from qigong and Dohsa-hou to yoga and aerobic exercise, using diverse research methodologies implemented across a variety of study populations. These included individuals practicing mindfulness and physical activity during the pandemic, healthy university students, undergraduate students meeting screening criteria for mobile phone addiction, breast cancer survivors with post-surgical pain, and women screening positive for adult ADHD.

While the scholarship on display in our Research Topic clarifies points about regulatory mechanisms of meditative movements through several cognitive indices of performance change, future research remains necessary to precisely account for the comparative advantage of dynamic to static meditative components. Studies might also further investigate elements of consistency in the choice of movements among the approaches and their relationships with the psychometric tools and behavioral tasks that were chosen for executive functions (Dang et al., 2020). Although included in our scope, some meditative movement types like tai chi remain a point for research and comparison. We hope that this effort stimulates future studies on meditative movement practices and their relationships to physical and mental health.

## Author contributions

RK and H-JY contributed equally to the writing of this editorial article. All authors contributed to the article and approved the submitted version.
